# Does Caring for Parents Take Its Toll? Gender Differences in Caregiving Intensity, Coresidence, and Psychological Well-Being Across Europe

**DOI:** 10.1007/s10680-023-09666-3

**Published:** 2023-06-28

**Authors:** Elisa Labbas, Maria Stanfors

**Affiliations:** Centre for Economic Demography, Lund University, PO Box 7080, 220 07 Lund, Sweden

**Keywords:** Unpaid caregiving, Psychological well-being, Coresidence, Gender, Country comparison, SHARE, OLS

## Abstract

Given population ageing and the emphasis on in-home care, more working-age adults are facing the demands of providing unpaid care to the elderly with potential implications for their own well-being. Such effects likely vary across Europe because care is differently organized with a differing emphasis on public support, dependence on family, and orientation toward gender equality. We studied the relationship between unpaid caregiving for elderly parents and the psychological well-being of older working-age (50–64) men and women by analysing data from the Survey of Health, Retirement, and Ageing in Europe (SHARE), covering 18 countries between 2004 and 2020 (N = 24,338), using ordinary least squares (OLS). We examined risk of depression by caregiving intensity and tested whether coresidence mediated outcomes. Men and women providing care to parents experience important psychological well-being losses across Europe, especially when caregiving is intensive. A heavier caregiving burden associated with coresidence explains a regime gradient in depression, not least for women in Southern Europe. Results highlight the spillover costs of unpaid caregiving across Europe and the need to address caregiver psychological well-being, especially in contexts where state support for elder care is low and coresidence is common.

## Introduction

Population ageing puts pressure on pensions and healthcare systems, creating an imperative to contain costs including those of Long-Term Care (LTC). Since the early 2000s, policymakers across Europe have emphasized in-home care over institutionalized care (Colombo et al., 2011, pp. 296–297). This implies that more adults are facing the demands of providing unpaid care to elderly and sick relatives. Despite unpaid care being the backbone of LTC systems, its indirect costs remain largely invisible. Many carers, not least women who provide more unpaid care than men, experience significant financial, social, and psychological strain from their caregiving role (especially when intensive) as it can crowd out paid work, leisure, and social interaction, or be otherwise detrimental to health and well-being (Al-Janabi et al., 2019; Carmichael & Charles, 2003; Pinquart & Sörensen, 2003; Van Houtven et al., 2019). All European nations will need to increase labour supply in the coming years, especially among older workers (50 +) and women, though it is unclear how unpaid care needs will impact this. It is thus vital to establish environments that keep caregivers in good health. For this, we need to understand and address the spillover effects of caregiving on working-age individuals for the sake of their well-being and regarding potential macroeconomic losses (e.g., lost productivity and sickness absence).

Comparing outcomes across countries may help us establish which environments reduce the well-being costs to the individual giving care. In Europe, countries are characterized by similar demographic ageing contexts but differ in their organization of elder care. There are differences in public support for those with care needs and for those providing unpaid care, and such support correlates with gender equality (Bettio & Plantenga, 2004). Differences in caregiver psychological well-being across Europe have been attributed to the availability of formal care and to cultural factors common to regional country clusters along a North–South divide (Brenna & Di Novi, 2016; Di Novi et al., 2015; Verbakel, 2014), but knowledge about specific mechanisms is lacking. For example, while adverse health effects may depend on caregiving intensity (Bremer et al., 2015) and the closeness in the relationship between caregiver and care recipient (Litwin et al., 2014), the role of coresidence (i.e., living with the care recipient) in caregiver psychological well-being has been little studied from a comparative perspective. One exception is a study by Kaschowitz and Brandt (2017). It does not, however, distinguish between older spousal caregivers and midlife individuals providing unpaid care to parents, and this distinction is highly important given the type of caregiving and its consequences. In Europe, coresidence of elderly persons with their adult children has declined and is common primarily in contexts where public support for elder care is low, and where much of the caregiving falls on family members, as in Southern Europe. In such contexts, traditional gender norms still structure the household division of labour, with women doing more unpaid work than men. Therefore, coresidence with elderly parents is a potential contributor to regional as well as gender differences in caregiver psychological well-being among midlife individuals, a subject yet to be investigated.

We examined how unpaid caregiving for elderly parents relates to the psychological well-being of older working-age (50–64) men and women across Europe, and addressed the following questions: (1) How does unpaid caregiving for parents and caregiving intensity relate to the psychological well-being of midlife men and women across Europe? (2) To what extent does coresidence of midlife adults and their parents explain a potential welfare state gradient in caregiver well-being? We used data from the Survey of Health, Retirement, and Ageing in Europe (SHARE), 2004–2020, covering 18 countries across Europe until the start of the COVID-19 pandemic. We estimated Ordinary Least Squares (OLS) models for average differences in the EURO-D measure of mental health intended to capture the risk of depression with a screening instrument for depressive symptoms.

The present study extends the literature on unpaid care and caregiver psychological well-being by exploring the spillover costs of unpaid care among the highly policy-relevant group of older working-age individuals, including both men and women, from a comparative perspective in the context of varying welfare state support for elder care. Focusing on those who provide unpaid care to elderly parents (while not confusing this type of caregiving with spousal care) is important and justified in that the prevalence of coresidence with elderly parents varies between countries whereas almost all spouses everywhere live together. The results provide novel insights regarding gender, unpaid care to parents and psychological well-being across Europe. They provide evidence of the role of caregiving intensity and coresidence in determining the welfare state regime gradient in caregiver depression. Results highlight the spillover costs of unpaid caregiving across Europe and the need to address caregiver psychological well-being, especially in contexts where state support for elder care is low and coresidence is common.

## Theoretical Considerations, Previous Research, and Hypotheses

### Caregiver Psychological Well-Being

While generating great social value, unpaid care has spillover costs borne by individual caregivers. According to the psychosocial stress perspective (Pearlin et al., 1990), caregiving impacts well-being directly by involving heavy physical tasks and emotional distress, and indirectly by creating stressful tension with other activities. Caregiving is associated with adverse health and well-being outcomes (see Bauer & Sousa-Poza, 2015; Bom et al., 2019; Pinquart & Sörensen, 2003; Van Houtven et al., 2019 for reviews). Though some studies find that caregiving is associated with positive experiences such as fulfilment, negative outcomes dominate. Carers report lower levels of subjective well-being, quality of life, and happiness (Bremer et al., 2015; Van den Berg et al., 2014). These negative experiences are especially salient when care is intensive (Coe & Van Houtven, 2009); when the parties involved are closely related (Litwin et al., 2014); and when caregiver and care recipient live together (Kaschowitz & Brandt, 2017)—probably because in-household care is typically more intensive and leads to feelings of always being ‘on duty’.

The “stress process” (Pearlin et al., 1990, p. 586) implies that the well-being impacts of caregiving are influenced by individual and contextual factors such as age and gender, caregiving intensity, and public or private support functions. Unpaid caregiving imposes on carers a trade-off with other activities, such as paid work and leisure. Although employed carers typically provide low levels of hands-on care, this implies both time costs and trade-offs. Not having enough time to perform all the tasks at hand or to recover results in stress (Hamermesh & Lee, 2007) and role conflict (Opree & Kalmijn, 2012; Stephens et al., 2001), and can result in taking sick leave (Ugreninov, 2013) or antidepressants and tranquilizers (Schmitz & Stroka, 2013).

The individual’s subjective perception of a given stressor (caregiving) and thus the stress experienced can vary depending on context and the social support available (Pearlin et al., 1990). Gender is a key factor in both caregiving and the stress process. In line with theories of time allocation and specialization within the household (Becker, 1965), women are more involved in unpaid caregiving than men across the life course. The moral and social obligation to provide care is stronger for women than men. In practice, daughters respond differently and more strongly to their parents’ needs compared to sons, even when they share socioeconomic traits (e.g., education, employment status, Haberkern et al., 2015). Women often fit caregiving into their schedule without cutting back on other family obligations by, for example, reducing leisure time (Stanfors et al., 2019). More extensive and frequent care responsibilities and greater care-work conflict impose a higher burden on women than on men.

### Caregiving Across Europe

The cross-national variation in state support for elder care can be conceptualized in terms of a welfare state and gender regime framework. Seen from this perspective, public policies are instruments that reduce the individual's dependency on the family or the market for ensuring the welfare (Esping-Andersen, 1999). The state can modify the caring function of the family by applying legal frameworks and LTC policy mixes ranging from familialistic to defamilializing. Welfare states generally support those in need of care through services or financial support, and caregivers receive support through leave schemes (with or without compensation) (Bettio & Plantenga, 2004; Leitner, 2003). Publicly provided or subsidized care services act as direct substitutes for unpaid care (Schmid et al., 2012). By alleviating individuals, especially women, of unpaid care responsibilities, welfare states have the potential to promote gender equality by supporting the female labour supply. Empirical studies focused on the organization of welfare production and gender equality often divide Europe into four country clusters: the Nordic countries, Continental Europe, the Southern European and the Central Eastern European (including the Baltic) countries (Daly, 2020, pp. 40–41).^1^ We acknowledge that this clustering is crude because heterogeneity exists among and within said clusters (Saraceno, 2016; Saraceno & Keck, 2011), but we nevertheless find it useful for comparative analysis.

The Nordic countries are the least oriented towards familialism because their policies have long been focused on minimizing the individual’s dependence on family. They are the most gender-egalitarian, having been early adopters of gender-neutral policies and the dual-earner/carer model. Most women are relieved of many care responsibilities for children and the elderly through the public organization of care. All adults are expected to work, while the state provides subsidized, universal access to care services and eases work-family conflict through paid leave schemes and reduced work hours for carers (Bettio & Plantenga, 2004). Such policies increase female employment and men’s involvement in unpaid activities. In a somewhat counterintuitive way, regular help and care provided by midlife adults to parents are more common (but less intensive) in the Nordic countries than elsewhere because state-provided services substitute the most structured and time-consuming care tasks (Brandt et al., 2009; Fokkema et al., 2008). Coresidence between adult children and their parents is almost non-existent.

Continental European countries are characterized by moderate, non-universal benefits and services. Countries such as France and Belgium feature fairly developed formal care strategies for dependents, while the Netherlands, Austria, and Germany expect families to coordinate care with public support (Bettio & Plantenga, 2004). The commitment to male breadwinning incentivizes a gendered division of labour. In sharp contrast to the Nordics, Southern European countries are the most familialistic in Western Europe. The state delegates responsibility for dependents to families via legal requirements, and the provision of benefits and services is low and means-tested. Gender structures men’s and women’s lives by enforcing a traditional division of labour and low female labour force participation. Coresidence with elderly parents is more common than elsewhere in Western Europe (Fokkema et al., 2008).

Eastern European countries have rarely been included in comparative analyses regarding unpaid care. Though egalitarianism and gender equality were strong, ideology-based features of their society, the transition to a market economy in the 1990s dismantled the welfare state which meant a reduction in publicly provided services in favour of market-based alternatives. Many women, especially in low-paying jobs, left the labour force due to the lack of care arrangements. While the Baltic and Central Eastern European countries still embrace gender equality (Daly, 2020, p. 40), familialism is widespread and the provision of formal elder care services limited (Saraceno & Keck, 2010). As in Southern Europe, coresidence remains relatively common (Treas & Cohen, 2006, p. 121).

Existing comparative studies on Europe suggest that there is a regional gradient in caregiver well-being among women from North to South that can be linked to differences in access to formal care as well as to social and cultural factors. However, such studies are few and vary in terms of method (e.g., how caregiving is defined, which carers and care recipients are studied). In their study on midlife women, Brenna and Di Novi (2016) found that caring for parents was positively related to depression in Southern Europe, though this was based on pooling caregiving both within and outside the household. Regarding coresidence, Kaschowitz and Brandt (2017) found that caregiving within the household was associated with depression irrespective of whether the organization of care might be labelled family-based (e.g., Austria, France, Germany, Spain, Italy) or service-based (Denmark, Sweden, Switzerland, Netherlands). This suggests that coresidence could be a driving factor behind regime variation among midlife caregivers. The authors did not test this hypothesis, however, because they pooled together all caregivers above 50 (including spousal caregivers, who are different in many respects from those caring for elderly parents, not least in terms of coresidence and the caregiving burden). For subjective well-being, impacts of caregiving among midlife women varied by welfare state regime depending on the dimension of quality of life studied (i.e., control, autonomy, pleasure, or self-realization) (Di Novi et al., 2015). According to Verbakel (2014), not only was the subjective well-being gap between caregivers and non-caregivers smaller in countries where formal long-term care resources were generous, but also a familialistic culture alleviated well-being losses from high-intensity caregiving. Taken together, these show a relationship between caregiving and psychological well-being that is detrimental to caregivers and varies across Europe; it relates to access to formal care and is gendered.

### Hypotheses

Based on the psychosocial stress perspective and previous research in the field, we made the following conjectures**.** We expected unpaid care for parents to be associated with lower psychological well-being, particularly among those providing time-intensive care (H1). Due to varying degrees to which the state alleviates family members of care responsibilities, we expected differences in well-being to be quite small in the Nordic countries but salient in Southern and Eastern Europe, with Continental European countries falling somewhere in between (H2). Knowing that women are the primary care providers even when in paid work, we expected their exposure to both primary and secondary stressors of caregiving to be higher compared to men, leading to greater differences in well-being (H3). Finally, since previous literature has indicated that coresidential care is more intensive than care outside the household, we expected that caring for a coresident parent would be a greater burden than caring for a parent living independently. Because intergenerational coresidence varies across welfare state regimes, we hypothesized that care for coresident parents could explain a potential regime gradient in caregiver well-being, at least for women (H4).

## Data and Methods

### Data and Sample

We used data from SHARE, which provides nationally representative samples of persons over the age of 50 and allows for international comparisons of various social outcomes through its cross-nationally ex-ante harmonized design (Börsch-Supan et al., 2013). We exploited data from 2004 to 2020 covering 18 countries across Europe, but excluded observations collected after the start of the COVID-19 pandemic and in the third wave which was a retrospective survey. Countries were clustered into Nordic (Sweden, Denmark), Continental (Germany, Austria, Netherlands, France, Switzerland, Belgium, Luxembourg), Southern (Spain, Italy, Greece, Portugal), and Eastern countries (Poland, Czech Republic, Slovenia, Estonia, Croatia), reflecting welfare states and gender regimes that differ in their organization of care.

We restricted our sample to men and women aged 50–64 who were currently employed or employable (i.e., not yet retired, nor permanently sick or disabled, nor in education). The self-employed were excluded due to potential differences in underlying characteristics. The upper age cut-off was set at 65, a common statutory retirement age in Europe. We excluded respondents who had limitations in performing the (instrumental) activities of daily living, as such limitations indicate they were themselves dependent on others for help. All respondents had at least one living parent. Because we were interested in caregiving for parents (the most common caregiving configuration at ages 50–64) and coresidence, we excluded respondents providing care only for someone other than a biological parent. We also removed observations with missing information on important variables, leaving us with 24,338 observations.

### Variables

As regards psychological well-being, SHARE provides the number of depressive symptoms on a 12-step EURO-D scale. This is a common mental health measure capturing feelings of depression, pessimism, suicidality, feelings of guilt, trouble sleeping, little interest in things, irritability, changes in appetite, fatigue, inability to concentrate, inability to feel enjoyment, and tearfulness. Reporting more than three symptoms suggests a “case of depression” and is indicated by a binary variable, which was more straightforward to use than the number of symptoms: the caregiving coefficient is interpreted as the change in risk of depression (according to said screening instrument) for caregivers compared to non-caregivers.

The first explanatory variable indicates whether respondents had provided care (e.g., personal care, practical household help, or help with paperwork) during the previous 12 months. The second explanatory variable captures caregiving intensity, dividing respondents into non-caregivers, low-intensity caregivers (weekly or less often), and high-intensity caregivers (daily/almost daily). To capture coresidence, we constructed a variable indicating whether the respondent lived in the same household or building as the parent.

Gender is central to the analysis as a structural determinant of caregiving and psychological well-being and is included in the models as a binary variable. We controlled for age to net out individual ageing. Health was captured by a variable for self-rated health, ranging from ‘poor’ to ‘excellent’. In addition, we employed a variable indicating any chronic physical health conditions. Partnership (married or cohabiting) was controlled for, as a partner may provide emotional support and share household duties but can also contribute to a heavier workload for women. We controlled for household size, total number of children, and having a child under the age of 15 in the household. Employment status and work hours were controlled for with a variable for working full-time (at least 35 h per week), working part-time, and not employed. On the one hand, working for pay provides economic and social resources and respite from caregiving, but on the other it can also create stress and overload when combined with caregiving. We used the 1997 International Standard Classification of Education (ISCED-97) categories low, middle, and high to control for level of education because those with high educational attainment earn more and may outsource caregiving, and they are more likely to work in flexible jobs, which helps when accommodating caregiving in daily life. Those with low educational attainment are more likely to cut back on paid work in response to caregiving duties, resulting in economic distress, loss of identity, and isolation, symptoms that are correlated with lower levels of psychological well-being. Change over time was factored out by year dummies.

For sensitivity analysis, we added country dummies to examine whether country differences in unobserved factors, such as gender norms, policy, and welfare state arrangements, were driving our results. We also estimated models with controls for equivalized gross household income, measured in quintiles. According to the stress process model, income determines the resources available for managing caregiving stressors (e.g., the purchase of care services). In addition to analysing the EURO-D measure, we investigated subjective well-being in a similar manner, using the log-transformed values of the CASP-12 index as a dependent variable. The CASP-12 index is a measure of quality of life (control, autonomy, pleasure, and self-realization), with higher values indicating higher quality of life. Results from these analyses are in line with those obtained for risk of depression but are less clear and can be found in Appendix.

Sample characteristics are displayed in Appendix Table 5.

### Analytical Plan

We estimated linear models by country cluster for average effects of caregiving intensity on psychological well-being by first pooling men and women. The full model controlled for gender, a vector of controls including sociodemographic factors, self-rated health, family status and household size, and year-fixed effects. Standard errors were clustered at respondent level for independence of estimates.

1 $${\text{Well}}\text{-}{\text{being}}_{i} = \alpha + \beta_{1} Caregiving_{i} + \beta_{2} Coresidence_{i} + \beta_{3} Gender + \beta_{4} {\mathbf{X}}_{i} + \beta_{5} Year_{i} + \varepsilon_{i}$$

Because men and women still differ in terms of caregiving and its sociodemographic determinants and the interactions arising therefrom, we stratified the analysis of caregiving intensity and coresidence by gender. The interaction effect shows the additional caregiving intensity effect, if any, for those coresiding with the parent(s) in their care. This allowed us to see whether there are significant differences in psychological well-being depending on caregiver coresidence.

2 $${\text{Well}} \text{-} {\text{being}}_{i} = \alpha + \beta_{1} {\text{Caregiving}}_{i} + \beta_{2} {\text{Coresidence}}_{i} + \beta_{3} {\text{Caregiving}} \times {\text{Coresidence}}_{i} + \beta_{4} {\mathbf{X}}_{i} + \beta_{5}\, {\text{Year}}_{i} + \varepsilon_{i}$$

Some studies employ individual-fixed effects models (FEMs) to reduce selection bias from unobserved, time-invariant characteristics (Kaschowitz & Brandt, 2017). Since FEMs rely on intra-individual variation, the statistical power of the analysis rests on how many people change caregiver status during the observation period. Despite a longitudinal component, SHARE data are limited, and the data we used were insufficient to estimate caregiving impacts by intensity or to test for interaction effects with coresidence. We nevertheless estimated FEMs for any caregiving separately for men and women. Results from these estimations can be found in Appendix (Table 10).

## Results

### Descriptive Results

The weighted proportions of caregiving for an elderly parent by country cluster, gender, intensity of care, and coresidence reflect the known paradoxical relationship between welfare state regimes and caregiving (Table 1). Providing any care is most common in the Nordic countries, where the state has the primary responsibility for securing the individual’s welfare; this is followed by Continental Europe and is least common in familialistic Southern and Eastern Europe. In contrast, the proportion providing high-intensity care is highest in Southern and Eastern Europe and markedly low in the Nordic countries. Furthermore, providing high-intensity care for a coresident parent is more common in these contexts. Slightly less than half of all high-intensity care is provided to a coresident parent in Southern and Eastern Europe, illustrating the greater role of the family in meeting elder care needs in less generous welfare state regimes.

**Table 1:** Weighted proportions (%) of caregiving to elderly parent(s) in Europe 2004–2020, by country cluster, gender, caregiving intensity, and coresidence. Significant gender differences in shares indicated with stars *Source*: Survey of Health, Ageing and Retirement in Europe (SHARE) (http://www.share-project.org/home0.html), waves 1–2 and 4–8 (excluding observations collected after the start of the COVID-19 pandemic)

	Nordic		Continental		South		East	
	Men	Women	Men	Women	Men	Women	Men	Women
Share providing any care	43 (538)	49** (945)	28 (1152)	37*** (2328)	17 (353)	30*** (971)	17 (365)	30*** (765)
*Of which*								
Low intensity	92 (509)	96 (892)	87 (1003)	79*** (1814)	62 (215)	49** (438)	65 (228)	63 (513)
High intensity	8 (29)	4 (53)	13 (149)	21*** (514)	38 (138)	51** (533)	35 (137)	37 (252)
*Of which*								
No coresidence	92 (23)	79* (40)	53 (88)	62 (342)	33 (48)	53** (225)	42 (65)	54 (126)
Coresidence	8 (6)	21* (13)	47 (61)	38 (172)	67 (90)	47** (308)	58 (72)	45 (126)
N	1396	2032	4523	6675	2124	3486	1713	2389

Any care defined as any regular help or care over the past 12 months. Low-intensity caregiving defined as weekly or less often, high-intensity caregiving defined as daily or almost daily. Coresidence defined as living in the same household or building as parent(s). Country clusters are Nordic: Sweden, Denmark; Continental: Germany, Austria, Netherlands, France, Switzerland, Belgium, Luxembourg; South: Spain, Italy, Portugal, Greece; East: Poland, Czech Republic, Slovenia, Estonia, and Croatia. Weighted using calibrated individual cross-sectional weights. Unweighted number of observations in parentheses

***p < 0.01, **p < 0.05, *p < 0.10

Women are disproportionately involved in caregiving across Europe. Even in the gender-equal Nordic countries, women are more likely to provide any care than men and to provide high-intensity care in Continental and Southern Europe. Caregiving duties are distributed more equally between genders in the Nordic and Eastern regimes, as is caregiving intensity. Among high-intensity caregivers in the South, the proportion coresiding with a parent is higher among men than women, possibly due to men’s primary role as providers of economic support (e.g., housing) rather than hands-on care. Taken together, women are more at risk of adverse well-being outcomes due to their greater role overall in caregiving. There may be significant differences in caregiver well-being across Europe depending on the welfare state arrangements for elder care and caregiver support.

Table 2 documents the weighted proportions of respondents at risk of depression (according to the EURO-D screening instrument) by gender, country cluster, caregiver status, and caregiving intensity. Caregiving correlates with psychological well-being among both men and women, although the correlations and statistical significance levels vary. The difference between caregivers and non-caregivers is usually more pronounced for high-intensity caregiving (H1). Women providing unpaid care to coresident parents in Southern and Eastern Europe display the highest risk of depression compared to other groups (H2). The risk of depression is also very high among Nordic men providing coresidential care, although only six men fall into this category (see Table 1). These findings suggest that the higher caregiving burden falling primarily on women in familialistic welfare state regimes plays a role in their well-being.

**Table 2:** Weighted proportions (%) of risk of depression (three or more depressive symptoms as indicated by EURO-D) by gender, country cluster, caregiver status and intensity, and coresidence *Source*: see Table 1

			By caregiving intensity			
	No care	Any care	Low intensity	High intensity		N
				No coresidence	Coresidence	
*Men*						
Nordic	6	10	9	19	100***	1396
Continental	14	17	16	25*	30	4523
South	12	14	15	15	13	2124
East	20	22	25	11	22	1713
*Women*						
Nordic	17	20	19	24	19	2032
Continental	26	25	23	36**	32	6675
South	28	38***	38**	32	45***	3486
East	30	41**	35	43	68***	2389

Significant differences between caregivers and non-caregivers indicated with stars

Weighted using calibrated individual cross-sectional weights

***p < 0.01, **p < 0.05, *p < 0.10

### Multivariate Results

Results from models stratified by country cluster provide support for our first hypothesis by showing that unpaid caregiving for parents is associated with lower psychological well-being across Europe (Table 3, Panel A). The coefficients for risk of depression follow a pattern corresponding with our expectations that the welfare state regime framework and caregiving burden fall on older working-age individuals (see H2). Coefficients are significant in the South (*β* = 0.06, *p* < 0.01) and East (*β* = 0.06, *p* < 0.01), modest in Continental Europe (*β* = 0.03, *p* < 0.01), but insignificant in the Nordics. At baseline, women are significantly more likely than men to be at risk of depression, irrespective of context, and coresidence is unrelated to the risk.

**Table 3:** OLS estimates of providing unpaid care to elderly parent(s) and risk of depression (three or more depressive symptoms as indicated by EURO-D) in Europe 2004–2020, by country cluster *Source*: see Table 1

A Any care	Nordic	Continental	South	East
*No care (ref.)*				
Any care	0.018 (0.013)	0.027*** (0.009)	0.056*** (0.014)	0.056*** (0.015)
*No coresidence (ref.)*				
Coresidence	− 0.011 (0.056)	− 0.021 (0.018)	0.008 (0.017)	− 0.002 (0.019)
*Man (ref.)*				
Woman	0.086*** (0.013)	0.109*** (0.009)	0.111*** (0.012)	0.085*** (0.013)
Constant	0.13	0.07	0.07	0.09
*R*^2^	0.10	0.10	0.15	0.12
B By caregiving intensity				
*No care (ref.)*				
Low intensity	0.012 (0.013)	0.019** (0.009)	0.043** (0.017)	0.041** (0.017)
High intensity	0.125*** (0.047)	0.066*** (0.019)	0.073*** (0.021)	0.087*** (0.025)
*No coresidence (ref.)*				
Coresidence	− 0.057 (0.054)	− 0.036* (0.019)	0.001 (0.018)	− 0.014 (0.020)
*Man (ref.)*				
Woman	0.086*** (0.013)	0.108*** (0.009)	0.111*** (0.012)	0.085*** (0.013)
Constant	0.13	0.07	0.07	0.09
*R*^2^	0.10	0.10	0.15	0.12
N	3428	11,198	5610	4102

All models control for age, self-rated health, chronic health condition, partnership, household size, child under age 15, number of children, employment status and full-time vs. part-time work, education, and survey year. Standard errors clustered at individual level in parentheses

***p < 0.01, **p < 0.05, *p < 0.10

As expected, caregiving intensity is important for understanding psychological well-being. High-intensity caregiving is associated with a higher depression likelihood across Europe (Table 3, Panel B), yet this applies more in the Nordic countries (*β* = 0.13, *p* < 0.01) and the East (*β* = 0.09, *p* < 0.01) than in the Continental and Southern (*β* = 0.07, *p* < 0.01) European countries. This contradicts the expectation of a welfare state regime gradient from North to South based on which countries provide more or less support for those needing care and those providing unpaid care. It might reflect the familialistic culture in the South affecting caregiver experience or, by the same token, the high degree of reliance on the state in the Nordic countries for care recipient support. The negative differences in caregiver well-being may reflect care recipients’ poor health and greater dependence on their children as well as challenges in reconciling caregiving with other areas of life, not least in contexts where high-intensity caregiving for parents is rare and carers have not expected it. In sum, high-intensity caregiving relates to considerably lower well-being in terms of depression risk across Europe, including the Nordic countries.

These results are robust to adding country-fixed effects, which account for unobserved factors at the country level. This implies that the patterns we observe regarding risk of depression are general and not driven by any country-specific factors. Of note, the results are also robust to the addition of household income controls. This suggests that household economic resources do not ease any negative well-being gaps.

We performed a gender-stratified analysis (Table 4) and found that while a positive association commonly exists between caregiving and depression risk for both men and women, patterns regarding caregiving intensity and coresidence differ. Model 1 provides baseline estimates. Model 2 includes an interaction between caregiving intensity and coresidence to test for significant differences according to coresidence for those providing high-intensity care (i.e., daily/almost daily).^2^ The coefficients show the impact of one variable when the other variable in the interaction is zero. The base effect of caregiving intensity indicates the association for those not living with their parents (reference category for coresidence). The coresidence coefficient shows the difference in depression risk among those not providing unpaid caregiving. The interaction effect shows the additional caregiving effect, if any, for those coresiding with parent(s). To find the net effect of caregiving (intensity) for coresident carers, the base and interaction effects must be added.

**Table 4:** OLS estimates of providing unpaid care to elderly parent(s) and risk of depression (three or more depressive symptoms as indicated by EURO-D), with interaction of high-intensity caregiving and coresidence shown *Source*: see Table 1

	Nordic		Continental		South		East	
	M1	M2	M1	M2	M1	M2	M1	M2
*Men*								
*No care (ref.)*								
Low intensity	0.002 (0.015)	0.002 (0.015)	0.023* (0.013)	0.023* (0.013)	0.034 (0.025)	0.035 (0.025)	0.062** (0.028)	0.062** (0.028)
High intensity	0.169** (0.079)	0.156* (0.086)	0.063* (0.037)	0.087* (0.049)	0.052 (0.035)	0.105* (0.057)	0.075** (0.038)	0.078 (0.053)
*No coresidence (ref.)*								
Coresidence	0.001 (0.089)	− 0.025 (0.096)	− 0.067*** (0.025)	− 0.055** (0.026)	− 0.037 (0.023)	− 0.025 (0.024)	− 0.002 (0.028)	− 0.001 (0.031)
*No care×No coresidence (ref.)*								
High intensity×Coresidence		0.085 (0.218)		− 0.071 (0.068)		− 0.092 (0.071)		− 0.006 (0.078)
Constant	0.09	0.10	0.09	0.09	0.09	0.09	0.10	0.10
*R*^﻿*2*^	0.08	0.08	0.08	0.08	0.11	0.11	0.12	0.12
N	1396		4523		2124		1713	
*Women*								
*No care (ref.)*								
Low intensity	0.017 (0.019)	0.017 (0.019)	0.015 (0.012)	0.015 (0.012)	0.045** (0.023)	0.043* (0.022)	0.031 (0.022)	0.030 (0.022)
High intensity	0.119** (0.059)	0.088 (0.065)	0.060*** (0.022)	0.069*** (0.025)	0.076*** (0.025)	0.036 (0.033)	0.089*** (0.033)	0.073 (0.046)
*No coresidence (ref.)*								
Coresidence	− 0.110 (0.069)	− 0.205*** (0.067)	− 0.004 (0.027)	0.014 (0.035)	0.019 (0.024)	− 0.011 (0.028)	− 0.022 (0.029)	− 0.034 (0.032)
*No care×No coresidence (ref.)*								
High intensity×Coresidence		0.215 (0.144)		− 0.045 (0.055)		0.093* (0.050)		0.042 (0.068)
Constant	0.25	0.25	0.18	0.18	0.18	0.19	0.17	0.17
*R*^2^	0.10	0.10	0.10	0.10	0.13	0.14	0.11	0.11
N	2032		6675		3486		2389	

All models control for age, self-rated health, chronic health condition, partnership, household size, child under age 15, number of children, employment status and full-time vs. part-time work, education, and survey year. Standard errors clustered at individual level in parentheses

***p < 0.01, **p < 0.05, *p < 0.10

Starting with the results for men, baseline estimates show that caregiving generally is associated with a higher depression risk in Continental and Eastern Europe. Only high-intensity caregiving is associated with depression risk in the Nordic countries, and there is no statistically significant association between caregiving and depression risk, net of observables, among men in the South. Coresidence is not important for depression risk, except in Continental Europe where it reduces the risk among those not providing care. Model 2 estimates indicate that caregiving, especially if high intensity, is associated with higher depression risk among men not living with their parents across Europe, including the South but excluding the East. There is a statistically significant difference according to coresidence status in Continental Europe for men not providing care. The interaction terms indicate no additional caregiver effect for men providing high-intensity care to a coresident parent. In the Nordic countries, the interaction is positive, suggesting that coresidential caregivers run a higher risk of depression than non-coresidential high-intensity caregivers, but it is not significant. In sum, coresidence does not explain increased depression risk among male caregivers.

Among women, baseline estimates show that low-intensity caregiving is associated with higher depression risk in the South, while high-intensity caregiving is important across all four regimes. Coresidence is not important for depression risk among those not providing care. Model 2 estimates indicate that caregiving is associated with higher depression risk among those not living with their parents in Continental and Southern Europe. Coresidence is important for psychological well-being only in the Nordics where coresidence is rare and it substantially reduces the risk of depression for those not providing care. The interaction terms indicate an additional caregiver effect for women in Southern Europe providing high-intensity care to a coresident parent. The interaction reduces the coefficient for high-intensity caregiving (in M1) to render it insignificant. A similar although not significant pattern arises in the Nordic countries. For women in Southern Europe, the interaction absorbs much of the positive association between high-intensity caregiving and depression risk. This provides support for the hypothesis that coresidential caregiving drives welfare state regime variation in well-being (H4), especially among women who tend to be the primary caregivers (H3). The results remain stable after adding country-fixed effects and controls for household income.

The findings from cross-sectional analyses should be viewed in the light of potential selection effects. Economic theory predicts that individuals with lower opportunity costs are more likely to engage in caregiving, particularly high-intensity caregiving (i.e*.*, negative selection). Further, high-income individuals can afford market substitutes that allow them to opt out of (high intensity) caregiving. Contrary to theoretical predictions, both positive (low-intensity caregiving) and negative (high-intensity caregiving) selection dynamics appear to be at play. This can lead to that cross-sectional methods over- or underestimate the well-being losses from caregiving. To reduce bias from unobserved characteristics correlating with socioeconomic characteristics and the propensity to provide care, we estimated FEMs for any caregiving separately for men and women. Few of the respondents changed in their caregiver status, which meant the data were insufficient to distinguish care by intensity or to test for interaction effects with coresidence. The FEM estimates nevertheless serve as selection indicators of relevance for psychological well-being. Interestingly, the results show that caregiving is associated with a significantly higher risk of depression among women in the Nordic countries. This goes against our theoretical expectations and suggests that caregiver well-being is dependent on context. The finding can be understood in the light of women’s high labour force participation and a “double burden” of working and caring, as well as cultural norms and expectations that public services (which are under strain) should shoulder most caregiving responsibilities (see Appendix Table 10).

## Discussion

This study draws attention to the indirect costs of unpaid caregiving rarely addressed in policy discourse. We focused on midlife men and women, who constitute an important part of the labour force in ageing nations but are also potential caregivers, not least for elderly parents. If the combination of work and caregiving or the crowding out of work due to work-care incompatibility is stressful and gives rise to social isolation or other negative experiences, this will likely affect working-age caregivers’ psychological well-being. Our initial findings are in line with previous studies that link caregiving to a deterioration in mental health (Bom et al., 2019; Pinquart & Sörensen, 2003), especially when caregiving is time-intensive (Van Houtven et al., 2019) and coresidential (Kaschowitz & Brandt, 2017).

We find important regional differences regarding unpaid caregiving and psychological well-being that reflect the types of welfare state and gender regime, which vary in terms of state support for care recipients and caregivers. The data allowed us to examine the role of coresidence in depression risk, given that living with and caring for an elderly parent is taxing but also much more common in contexts where the family rather than the state bears the primary responsibility for elder care. Previous country comparisons of caregiver psychological well-being have attributed variation in outcomes to the availability of formal care as well as cultural norms surrounding care, especially for women. Our study expands on this literature by showing that well-being losses are obvious in a context where coresidence with elderly parents is common, namely Southern Europe. These findings are in line with the theoretical expectation that greater responsibility placed on the family for elder care means higher exposure to the stressors of caregiving. The findings corroborate previous research on depression and in-household caregiving (Kaschowitz & Brandt, 2017) and show that coresidence between elderly parents and their adult children should be considered in future country-comparative studies.

Care responsibilities have different impacts on men and women. Our findings suggest these are more likely to be negative for women than for men (not least because women are also more likely to provide high-intensity care). In addition, we found that coresidence explains the welfare state gradient in caregiver well-being among women. This highlights the importance of traditional gender roles in that women have the main responsibility for care and perform heavy and routine care tasks, whereas men engage in less demanding tasks that can also be more easily fitted into their schedule. That coresidence explains welfare state variation in well-being losses reflects how the indirect costs of elder care in familialistic country contexts are borne by adult daughters. It also suggests that greater support for those in need of care can alleviate well-being losses for caregivers and support women’s well-being and employment. Nevertheless, men are also at risk of adverse well-being outcomes and should not be neglected in future research or policymaking, especially given an increasingly ageing population and the increasing importance attached to men’s caregiving contributions.

Our findings are cross-sectional, and they could neither rule out the influence of omitted factors nor claim causality. This means that while caregivers are more likely to experience lower psychological well-being than non-caregivers, differences can reflect the socioeconomic factors and policy environment putting them at a disadvantage rather than reflect the caregiving itself. More research is needed to separate effects of selection from effects of caregiving, including care for coresident parents. We would encourage those engaged in future research to extend the longitudinal data, given the potential to reduce selection bias.^3^

After accounting for selection by adding individual fixed effects, we found that caregiving relates to higher risk of depression among women in the Nordic countries. This contrasts with expectations because in the Nordics the state has primary responsibility for elder care, gender equality is high, and both women and men work full-time even in older age. The intensity of hands-on care provided by family members tends to be low as formal care personnel perform the heaviest tasks. Our findings suggest that while distress from hands-on care is lower than in more familialistic contexts, a different kind of caregiving burden exists in defamilialized contexts. A high degree of gender equality in the labour market means that women fit caregiving into their already full schedule, creating a “double burden” of paid work and caregiving, stress, and giving little time for rest and recovery. Our findings are in line with the view that a blind spot in elder care policy exists in terms of meeting the needs of working-age caregivers.

We did not attempt to disentangle the well-being effect of caregiving from the effect of having a frail or sick parent, which can have adverse health effects on adult children independently of caregiving (Amirkhanyan & Wolf, 2006; Bobinac et al., 2010). As this “family effect” often occurs simultaneously with caregiving, disentangling the two and making country and gender comparisons requires a much larger sample size than that available for our study.^4^ Although studies that have successfully separated these effects remain rare (Bom et al., 2019), Heger (2017) for one find that a separate caregiver effect on psychological well-being does exist. Our estimates can be interpreted as showing the combined effect of having an ailing parent and caregiving. Future studies might explore the contributions of the family effect and caregiving as more data becomes available.

Our study considers a particular group of caregivers, namely adults aged 50–64 who care for their own parent. This should be kept in mind because caregivers for parents can also be younger than 50. Including younger ages, however, would require a careful approach so as not to confound the impacts of caregiving for parents with those of caregiving for dependent children, and it would also require a more careful approach when investigating which working conditions allow for greater work-care compatibility than others. Furthermore, we only considered caregiving for one’s own parents. It may be interesting to investigate the well-being effects of caring for parents-in-law and to compare outcomes for caregivers for parents, given the different types of personal relationships.

We purposefully excluded observations collected after the start of the COVID-19 pandemic, considering it a separate research topic. The crisis exacerbated challenges for caregivers and shed light on longstanding challenges in the formal elder care sector, accelerating the public debate around the organization of care in many countries. The impact of the pandemic on caregiver well-being and implications for elder care policies remains a subject of future study.

Formal care policies can reduce the care load of families and mitigate adverse outcomes, especially in contexts where the current level of provision is low. Increasing the accessibility and quality of institutional and in-home care is particularly important because it offers options to those who care for severely disabled, coresident elderly family members. Social care policies reduce the amount of unpaid caregiving by women (Haberkern et al., 2015) and associated well-being losses. It is also important to address the needs of workers who have responsibilities towards elderly family members since mental health losses contribute to negative outcomes in the workplace. At present, families across Europe with dependent elderly members receive much less support than families with children and are largely ignored in work-family reconciliation policies. The lack of coherent regulation regarding leave schemes, flexible working time arrangements, or right to request reduced working hours means that carers in many contexts are left to rely on the benevolence of employers. It is thus important to establish adequate reconciliation measures to keep the larger share of older workers, especially women, in the labour force.

## Conclusion

In the context of population ageing, it is vital to understand the implications of the increasing care needs of families with elderly members and society at large. This study has produced new evidence of the spillover costs of providing unpaid care to elderly parents for the psychological well-being of older working-age (50–64) men and women across Europe. Our findings corroborate research showing that unpaid caregiving (especially if high-intensity) is on balance related to lower well-being; specifically, an increased risk of depression. They show that coresidence with elderly parents, which is most common in Southern Europe, ties into a welfare state regime gradient in caregiver depression risk. The increased risk in Southern Europe is limited to women and connected to traditional gender roles and different exposure to stressors. This reflects the fact that in contexts where public support for elder care is low, the extensive responsibility to ensure the welfare of elderly parents can leave adult children (daughters) with a high burden of care.

Care needs increase faster than public care services expand, and continued policy emphasis on in-home care will likely accentuate negative impacts on unpaid caregivers (Bobinac et al., 2010; Pinquart & Sörensen, 2003). To maintain workforce well-being and control healthcare costs, caregiver psychological well-being should be supported by targeted policies for the elderly with care needs *and* for the family members providing that care. These consist of (but are not limited to) adequate access to quality formal care services, particularly in countries where current provision is low, and support measures (e.g., respite care, financial support) for those caring for loved ones at home. It is important to note that a coherent implementation of care-work reconciliation measures (e.g., flexible work time, remote working, adapted work hours) would be needed. These measures could both unburden families of the need to provide care themselves and enable men and women to provide sporadic assistance and emotional support compatible with other obligations.

## Data Availability

This paper uses data from SHARE Waves 1, 2, 3, 4, 5, 6, 7 and 8 (DOIs: https://doi.org/10.6103/SHARE.w1.800, https://doi.org/10.6103/SHARE.w2.800, https://doi.org/10.6103/SHARE.w4.800, https://doi.org/10.6103/SHARE.w5.800, https://doi.org/10.6103/SHARE.w6.800, https://doi.org/10.6103/SHARE.w7.800, https://doi.org/10.6103/SHARE.w8.800, see Börsch-Supan et al. (2013) for methodological details. The SHARE data collection has been funded by the European Commission, DG RTD through FP5 (QLK6-CT-2001–00,360), FP6 (SHARE-I3: RII-CT-2006–062,193, COMPARE: CIT5-CT-2005–028,857, SHARELIFE: CIT4-CT-2006–028,812), FP7 (SHARE-PREP: GA N°211,909, SHARE-LEAP: GA N°227,822, SHARE M4: GA N°261,982, DASISH: GA N°283,646) and Horizon 2020 (SHARE-DEV3: GA N°676,536, SHARE-COHESION: GA N°870,628, SERISS: GA N°654,221, SSHOC: GA N°823,782, SHARE-COVID19: GA N°101,015,924) and by DG Employment, Social Affairs & Inclusion through VS 2015/0195, VS 2016/0135, VS 2018/0285, VS 2019/0332, and VS 2020/0313. Additional funding from the German Ministry of Education and Research, the Max Planck Society for the Advancement of Science, the U.S. National Institute on Aging (U01_AG09740-13S2, P01_AG005842, P01_AG08291, P30_AG12815, R21_AG025169, Y1-AG-4553–01, IAG_BSR06-11, OGHA_04-064, HHSN271201300071C, RAG052527A) and from various national funding sources is gratefully acknowledged (see www.share-project.org).

## Appendix

See Tables 5, 6, 7, 8, 9, 10.

**Table 5:** Sample characteristics: Weighted means and proportions (%) for all respondents and caregivers only by country cluster *Source*: Survey of Health, Ageing and Retirement in Europe (SHARE) (http://www.share-project.org/home0.html), waves 1–2 and 4–8 (excluding observations collected after the start of the COVID-19 pandemic)

	All				Low-intensity caregivers				High-intensity caregivers			
Caregiving	Nordic	Continental	South	East	Nordic	Continental	South	East	Nordic	Continental	South	East
No care	54	72	76	76								
Any care	46	33	24	24								
*Of which*												
Low intensity	94	82	53	64								
High intensity	6	18	47	36								
*Coresidence with parent*												
No coresidence	99	94	86	87	NA	NA	NA	NA	87	60	48	51
Coresidence	1	6	14	13	NA	NA	NA	NA	13	40	52	49
*Gender*												
Men	47	45	46	46	43	40	37	33	61	28	26	31
Women	53	55	54	54	57	60	63	67	39	72	74	69
*Age*												
50–54	34	44	46	46	31	43	48	53	32	34	34	41
55–59	46	42	41	43	49	44	40	37	53	45	45	48
60–64	20	13	13	10	20	13	12	9	15	21	21	11
*Self-rated health*												
Poor	1	2	2	5	1	1	2	3	0	2	1	4
Fair	8	14	16	17	7	12	15	13	6	18	21	21
Good	25	45	44	50	27	40	46	48	26	49	43	52
Very good	34	27	27	23	33	33	26	32	26	19	23	18
Excellent	33	11	11	5	32	14	10	5	41	11	11	5
*Physical health*												
No health condition	48	44	46	43	48	43	38	37	60	41	37	39
At least one health condition^a^	52	56	54	57	52	57	62	63	40	59	63	61
*Family characteristics*												
No partner	25	23	20	29	27	29	25	33	25	24	28	47
Partnered	75	77	80	71	73	71	75	67	75	76	72	53
Household size	2.21	2.41	2.97	2.64	2.20	2.31	2.79	2.47	2.38	2.40	2.90	2.44
No child under 15	93	93	93	96	92	94	93	97	88	95	98	100
Child under 15	7	7	7	4	8	6	7	3	12	5	2	0
Total number of children	2.09	1.79	1.52	1.88	2.17	1.80	1.56	1.81	2.39	1.67	1.45	1.70
*Employment and work hours*												
Employed full-time (35 h +)	77	58	50	68	77	59	48	81	69	39	35	60
Employed part-time (< 35 h)	17	23	14	8	17	25	39	9	23	31	20	11
Not in employment	6	18	36	24	5	16	33	9	8	30	46	29
*Education*												
Low	14	14	52	16	13	10	39	6	15	15	64	17
Middle	38	53	32	65	38	50	39	60	41	61	27	61
High	47	33	16	19	49	40	22	33	45	24	8	22
*Household income*												
Quintile 1	18	16	21	22	16	11	14	13	31	20	22	30
Quintile 2	20	18	19	15	20	16	16	12	9	19	21	12
Quintile 3	20	21	18	17	20	22	19	18	17	21	22	22
Quintile 4	20	22	21	18	22	23	25	23	29	20	20	19
Quintile 5	21	23	22	27	23	28	27	34	14	20	16	16
N	3,428	11,198	5,610	4,102	1,401	2,817	653	741	82	663	671	389

^a^Defined as a physical condition that the respondent has ever been diagnosed with (waves 1 and 5) or is currently suffering from (waves 2, 4 and 6–8), such as a heart attack or other cardiovascular condition, stroke or cerebral vascular disease, diabetes, chronic lung disease, cancer or malignant tumour, ulcers, Parkinson’s or Alzheimer’s disease, cataracts, arthritis, or hip fracture. Weighted using calibrated individual cross-sectional weights

**Table 6:** Weighted means of quality of life (as indicated by CASP-12) by gender, country cluster, caregiver status and intensity, and coresidence *Source*: See Table 5

			By caregiving intensity			
	No care	Any care	Low intensity	High intensity		N
				No coresidence	Coresidence	
*Men*						
Nordic	3.71	3.70*	3.70	3.70	3.60	1,396
Continental	3.67	3.66	3.66	3.62	3.66	4,523
South	3.60	3.56**	3.56**	3.57*	3.55	2,124
East	3.59	3.62*	3.60	3.67**	3.68*	1,713
*Women*						
Nordic	3.71	3.70	3.70	3.70	3.67	2,032
Continental	3.67	3.68	3.68**	3.65	3.64	6,675
South	3.58	3.57	3.58	3.56	3.54**	3,486
East	3.60	3.57	3.59	3.56	3.53	2,389

Significant differences between caregivers and non-caregivers indicated with stars

Weighted using calibrated individual cross-sectional weights

****p* < 0.01, ***p* < 0.05, **p* < 0.10

**Table 7:** OLS estimates of providing unpaid care to elderly parent(s) and quality of life (as indicated by CASP-12) in Europe 2004–2020, by country cluster *Source*: see Table 5

A Any care	Nordic	Continental	South	East
*No care (ref.)*				
Any care	− 0.011*** (0.004)	− 0.012*** (0.003)	− 0.007 (0.005)	− 0.025*** (0.006)
*No coresidence (ref.)*				
Coresidence	0.015 (0.015)	0.004 (0.006)	0.008 (0.007)	0.000 (0.008)
*Man (ref.)*				
Woman	0.005 (0.004)	0.001 (0.003)	0.003 (0.005)	− 0.001 (0.005)
Constant	3.68	3.69	3.61	3.65
*R*^*2*^	0.15	0.16	0.16	0.17
B By caregiving intensity				
*No care (ref.)*				
Low intensity	− 0.011*** (0.004)	− 0.008*** (0.003)	− 0.003 (0.007)	− 0.022*** (0.006)
High intensity	− 0.021* (0.011)	− 0.027*** (0.007)	− 0.011 (0.008)	− 0.032*** (0.010)
*No coresidence (ref.)*				
Coresidence	0.019 (0.015)	0.010 (0.006)	0.010 (0.007)	0.003 (0.009)
Man *(ref.)*				
Woman	0.005 (0.004)	0.001 (0.003)	0.003 (0.005)	− 0.001 (0.005)
Constant	3.68	3.69	3.61	3.65
*R*^*2*^	0.15	0.16	0.16	0.17
N	3,428	11,198	5,610	4,102

All models control for age, self-rated health, chronic health condition, partnership, household size, child under age 15, number of children, employment status and full-time vs. part-time work, education, and survey year. Standard errors clustered at individual level in parentheses

****p* < 0.01, ***p* < 0.05, **p* < 0.10

**Table 8:** OLS estimates of providing unpaid care to elderly parent(s) and quality of life (as indicated by CASP-12), by country cluster and gender *Source*: see Table 5

	Nordic	Continental	South	East
*Men*				
*No care (ref.)*				
Any care	− 0.002 (0.006)	− 0.012** (0.005)	− 0.022** (0.010)	− 0.021** (0.009)
*No coresidence (ref.)*				
Coresidence	− 0.013 (0.032)	0.029*** (0.008)	0.012 (0.011)	0.015 (0.012)
Constant	3.68	3.67	3.58	3.63
*R*^2^	0.13	0.19	0.13	0.19
*N*	1,396	4,523	2,124	1,713
*Women*				
*No care (ref.)*				
Any care	− 0.017*** (0.005)	− 0.011*** (0.004)	− 0.002 (0.006)	− 0.028*** (0.007)
*No coresidence (ref.)*				
Coresidence	0.031** (0.013)	− 0.014 (0.009)	0.004 (0.008)	− 0.010 (0.011)
Constant	3.68	3.70	3.63	3.66
*R*^2^	0.19	0.15	0.19	0.16
N	2,032	6,675	3,486	2,389

See Table 7

****p* < 0.01, ***p* < 0.05, **p* < 0.10

**Table 9:** OLS estimates of providing unpaid care to elderly parent(s) and quality of life (as indicated by CASP-12), baseline model (M1) and model including interaction of high-intensity caregiving and coresidence (M2) *Source*: see Table 5

	Nordic		Continental		South		East	
	M1	M2	M1	M2	M1	M2	M1	M2
*Men*								
*No care (ref.)*								
Low intensity	− 0.001 (0.006)	− 0.001 (0.006)	− 0.011** (0.005)	− 0.011** (0.005)	− 0.014 (0.011)	− 0.014 (0.011)	− 0.023** (0.011)	− 0.023** (0.011)
High intensity	− 0.019 (0.017)	− 0.009 (0.017)	− 0.018 (0.013)	− 0.019 (0.017)	− 0.037** (0.016)	− 0.047* (0.026)	− 0.017 (0.013)	− 0.002 (0.016)
*No coresidence (ref.)*								
Coresidence	− 0.007 (0.030)	0.015 (0.031)	0.031*** (0.009)	0.031*** (0.009)	0.015 (0.011)	0.013 (0.012)	0.014 (0.012)	0.021 (0.013)
*No care﻿×**No coresidence (ref.)*								
High intensity﻿×Coresidence		− 0.071 (0.063)		0.003 (0.024)		0.018 (0.033)		− 0.033 (0.028)
Constant	3.68	3.68	3.67	3.67	3.58	3.58	3.63	3.63
*R*^*2*^	0.13	0.13	0.19	0.19	0.13	0.13	0.19	0.19
N	1,396		4,523		2,124		1,713	
*Women*								
*No care (ref.)*								
Low intensity	− 0.016*** (0.005)	− 0.016*** (0.005)	− 0.007* (0.004)	− 0.007* (0.004)	0.002 (0.008)	0.003 (0.008)	− 0.022*** (0.008)	− 0.022*** (0.008)
High intensity	− 0.026* (0.014)	− 0.023 (0.016)	− 0.028*** (0.008)	− 0.032*** (0.009)	− 0.006 (0.009)	0.002 (0.011)	− 0.040*** (0.013)	− 0.040** (0.017)
*No coresidence (ref.)*								
Coresidence	0.036*** (0.014)	0.045*** (0.014)	− 0.006 (0.009)	− 0.014 (0.010)	0.006 (0.009)	0.012 (0.010)	− 0.004 (0.012)	− 0.004 (0.013)
*No care﻿×No coresidence (ref.)*								
High intensity﻿×Coresidence		− 0.021 (0.029)		0.019 (0.019)		− 0.018 (0.018)		− 0.000 (0.026)
Constant	3.68	3.68	3.70	3.70	3.63	3.63	3.66	3.66
*R*^2^	0.19	0.19	0.16	0.16	0.19	0.19	0.17	0.17
N	2,032		6,675		3,486		2,389	

See Table 7

****p* < 0.01, ***p* < 0.05, **p* < 0.10

**Table 10:** Individual-fixed effects estimates of providing unpaid care to elderly parent(s) and psychological well-being (as indicated by EURO-D and CASP-12), by country cluster﻿ *Source*: see Table 5

	Depression (EURO-D)				Quality of life (CASP-12)			
	Nordic	Continental	South	East	Nordic	Continental	South	East
*Men*								
*No care (ref.)*								
Any care	− 0.006 (0.021)	0.048** (0.019)	− 0.012 (0.037)	0.067 (0.046)	− 0.013* (0.007)	− 0.004 (0.006)	− 0.020 (0.014)	0.005 (0.014)
*No coresidence (ref.)*								
Coresidence	− 0.012 (0.062)	− 0.090 (0.055)	− 0.096* (0.055)	0.032 (0.081)	− 0.065** (0.027)	0.038** (0.017)	0.026 (0.025)	0.009 (0.027)
Constant	0.14	0.19	0.13	− 0.06	3.77	3.65	3.65	3.64
*R*^2^	0.08	0.04	0.06	0.11	0.08	0.07	0.12	0.09
N	1,396	4,523	2,124	1,713	1,396	4,523	2,124	1,713
*Women*								
*No care (ref.)*								
Any care	0.066** (0.029)	− 0.019 (0.019)	0.015 (0.028)	0.047 (0.035)	− 0.009* (0.005)	− 0.007 (0.005)	− 0.010 (0.009)	− 0.011 (0.009)
*No coresidence (ref.)*								
Coresidence	0.153 (0.122)	0.013 (0.060)	0.121** (0.049)	− 0.020 (0.054)	0.000 (0.026)	0.003 (0.013)	0.021 (0.015)	− 0.012 (0.016)
Constant	0.28	0.31	0.30	0.46	3.76	3.67	3.61	3.54
*R*^2^	0.04	0.05	0.08	0.06	0.09	0.04	0.10	0.06
N	2,032	6,675	3,486	2,389	2,032	6,675	3,486	2,389

See Table 7

****p* < 0.01, ***p* < 0.05, **p* < 0.10

## Footnotes

1 Beside the concepts of welfare state and gender regime, scholars also use the concept of care regime (Bettio & Plantenga, 2004; Naldini, Pavolini & Solera, 2016; Simonazzi, 2009) which focuses on the organization of care as opposed to the organization of welfare more generally. Instead of looking for common denominators, studies in this vein of research usually emphasize how policies differ between countries, thus challenging the validity of welfare state regimes for analysing the organization of care.

2 Low-intensity caregiving is not interacted with coresidence because it is provided to independently living care recipients.

3 Furthermore, studies using panel data could examine how the impact of caregiving varies for individuals over time, e.g., whether carers adjust to their situation.

4 SHARE includes information on parent health for most of our observations. Controlling for poor parent health reduces the caregiving effect, as expected (results made available per request). Because poor parent health and caregiving tend to coincide, the sample sizes are too small to infer country differences in the caregiver effect.
